# Negative contrast Cerenkov luminescence imaging of blood vessels in a tumor mouse model using [^68^Ga]gallium chloride

**DOI:** 10.1186/2191-219X-4-15

**Published:** 2014-03-08

**Authors:** Jeffrey D Steinberg, Anandhkumar Raju, Prashant Chandrasekharan, Chang-Tong Yang, Karen Khoo, Jean-Pierre Abastado, Edward G Robins, David W Townsend

**Affiliations:** 1 Singapore Bioimaging Consortium, Agency for Science, Technology and Research, Singapore, Singapore; 2 Singapore Immunology Network, Agency for Science, Technology and Research, Singapore, Singapore; 3 Clinical Imaging Research Centre, Agency for Science, Technology and Research - National University of Singapore, Singapore, Singapore

**Keywords:** Cerenkov luminescence imaging, [^68^Ga]gallium chloride, Tumor, Blood vessels, Negative contrast imaging

## Abstract

**Background:**

Cerenkov luminescence imaging (CLI) is an emerging imaging technique where visible light emitted from injected beta-emitting radionuclides is detected with an optical imaging device. CLI research has mostly been focused on positive contrast imaging for ascertaining the distribution of the radiotracer in a way similar to other nuclear medicine techniques. Rather than using the conventional technique of measuring radiotracer distribution, we present a new approach of negative contrast imaging, where blood vessel attenuation of Cerenkov light emitted by [^68^Ga]GaCl_3_ is used to image vasculature.

**Methods:**

BALB/c nude mice were injected subcutaneously in the right flank with HT-1080 fibrosarcoma cells 14 to 21 days prior to imaging. On the imaging day, [^68^Ga]GaCl_3_ was injected and the mice were imaged from 45 to 90 min after injection using an IVIS Spectrum *in vivo* imaging system. The mice were imaged one at a time, and manual focus was used to bring the skin into focus. The smallest view with pixel size around 83 μm was used to achieve a sufficiently high image resolution for blood vessel imaging.

**Results:**

The blood vessels in the tumor were clearly visible, attenuating 7% to 18% of the light. Non-tumor side blood vessels had significantly reduced attenuation of 2% to 4%. The difference between the attenuation of light of tumor vessels (10% ± 4%) and the non-tumor vessels (3% ± 1%) was significant. Moreover, a necrotic core confirmed by histology was clearly visible in one of the tumors with a 21% reduction in radiance.

**Conclusions:**

The negative contrast CLI technique is capable of imaging vasculature using [^68^Ga]GaCl_3_. Since blood vessels smaller than 50 μm in diameter could be imaged, CLI is able to image structures that conventional nuclear medicine techniques cannot. Thus, the negative contrast imaging technique shows the feasibility of using CLI to perform angiography on superficial blood vessels, demonstrating an advantage over conventional nuclear medicine techniques.

## Background

Recent advances in medical imaging have led to new advances using existing technologies. An example of this alternative use of existing technologies was the introduction of Cerenkov luminescence imaging (CLI) by Robertson et al. [1] and Spinelli et al. [2]. Robertson et al. demonstrated that with the introduction of super-cooled charge-coupled devices, it was possible to use optical imaging to determine the distribution of positron emission tomography (PET) radiotracers such as 2-deoxy-2-[^18^F]fluoro-d-glucose (FDG) *in vivo* due to the emission of Cerenkov light. Several groups have explored the possibilities of this new medical imaging technique, including the use of other radioisotopes and 3D reconstruction [3-5]. There have also been a few groups that have tested the technique in human subjects [6,7].

CLI has a few advantages over existing imaging technologies. Researchers have presented one advantage by demonstrating the feasibility of using CLI to study the efficacy of drug therapy [8,9]. They argue that CLI provides a cheaper, faster alternative to PET due to the ability to image five mice at the same time using an optical imaging device. Another advantage that CLI has is its ability to not only image the β^+^ emitting PET radiotracers, but also image β^−^ emitting particles [6,10].

CLI has mainly been used as an alternative method for imaging the distribution of PET tracers, but one can acquire new anatomical information from CLI due to its optical imaging properties. PET imaging detects the gamma rays that result from the annihilation of the emitted positron. The gamma rays pass through tissue and most materials, allowing one to detect the location of radiotracers deep within the body. CLI, on the other hand, relies on Cerenkov light, which is produced from positrons traveling faster than the speed of light within the tissue. Thus, CLI detects activity at the site of the decay of the radioisotope rather than the location of annihilation of the positron, reducing the issue of positron range, which limits the resolution of PET [11]. However, CLI is limited in its detection ability since the Cerenkov light gets attenuated and scattered by biological tissue, making imaging of internal structures more difficult.

In this paper, we evaluate a new approach to CLI using negative contrast imaging to image the light attenuated by the blood vessels. In order to image the trace number of photons attenuated by the blood, a radioisotope with high Cerenkov light output was needed for adequate sensitivity. Park et al. [12] and Beattie et al. [13] showed that ^68^Ga had much higher Cerenkov light output than ^18^F, making it an excellent candidate for negative contrast imaging. Moreover, the gallium ion binds to plasma proteins resulting in a thorough distribution throughout the body [14,15]. The magnitude and distribution of light in the body make [^68^Ga]GaCl_3_ an excellent compound for producing the background lighting necessary for negative contrast imaging.

## Methods

### [^68^Ga]GaCl_3_ production

[^68^Ga]GaCl_3_ was obtained from an ITG ^68^Ge/^68^Ga generator (Isotope Technologies Garching, GmbH, Germany) by elution with 10 mL 0.05 M HCl. After eluting and discarding the first 0.5 mL, the following 2 mL of [^68^Ga]GaCl_3_ was collected from the elution. The resulting [^68^Ga]GaCl_3_ (>99% radio purity) was neutralized using approximately 0.3 mL of 0.5 M NaOH to adjust pH to 7.0 ± 0.5, and the formulated sample had an activity of 300 to 400 MBq/mL at the time of receipt.

### Phantom study

[^68^Ga]GaCl_3_ was placed in plastic vials inside a well plate. Vials contained anywhere from 0 to 90 μL of ^68^Ga with saline added so that the total volume of each vial was 1 mL. The calculated activity of ^68^Ga in each vial ranged from 0 to 0.61 MBq, which was decay-corrected to the time of the scan. The phantom was scanned for 5 min on a Caliper Life Sciences IVIS Spectrum *in vivo* imaging system (PerkinElmer Life Sciences, Hopkinton, MA, USA). The average photon emission of each sample was measured after subtraction of cosmic radiation using a region of interest (ROI) centered on the vial using Living Image 4.3.1 software, and the radiance (p/s/cm^2^/sr) determined from the ROI was decay-corrected. Using the same method, the measurements were acquired using 0 to 7.44 MBq of [^18^F]FDG. The average radiance versus activity of the radiotracer was plotted for both radiotracers and fitted using a linear regression. The slope of the linear fit was used to determine the average radiance per activity for each radioisotope.

### Mouse model

All studies were approved by the Institutional Animal Care and Use Committee of the Biological Resource Centre. BALB/c nude mice were used in this study, and the tumors were derived from HT-1080 human fibrosarcoma acquired from the American Type Culture Collection (ATCC; University Boulevard, Manassas, VA, USA). The cells were cultured in Dulbecco’s modified Eagle’s medium supplemented with 10% fetal bovine serum and 1% penicillin-streptomycin in a humidified incubator with 5% CO_2_. Each mouse was injected subcutaneously with one million cells suspended in 100 μL saline (0.9% NaCl). The tumors were allowed to grow for 14 to 21 days reaching a diameter of 6 to 10 mm. The growth characteristics of the HT-1080 cell line in nude mice have been presented by several researchers [16-18].

### Biodistribution

Nine female nude mice of 8 to 9 weeks old bearing HT-1080 xenograft tumor were injected intravenously with 6 to 17 MBq in 100 μL of the formulated [^68^Ga]GaCl_3_. The animals were warmed up in an incubator at 36°C for 30 to 40 min before injection. After 30, 65, and 120 min post injection, a cardiac puncture using a needle and syringe was done for exsanguination while under anesthesia with oxygen and 2% isoflurane mixture. The euthanized mice were dissected, the organs were weighed, and the radiation in each organ was measured using a PerkinElmer gamma counter. Radioactivity in the organs was determined by calibrating the instrument with a standard curve with a known activity of ^68^Ga. The radioactivity was decay-corrected, and the results were expressed as percentage injected dose per unit weight of the organ (%ID/g). The biodistribution was also evaluated on an IVIS by placing the organs inside a well plate in the scanner. The organs were scanned for 5 min using view D with small binning (21.7 cm × 21.7 cm field of view, 480 × 480 pixels, 0.45 mm × 0.45 mm pixel size, f/stop = f/1).

### Positron emission tomography

In order to understand the *in vivo* behavior of [^68^Ga]GaCl_3_ radiotracer, a dynamic PET emission scan was performed on a Siemens Inveon Positron Emission Tomography/Computed Tomography (PET/CT) system (Siemens Healthcare USA, Inc., Malvern, PA, USA) over 120 min after injection of 100 μL (20 MBq) of the radiotracer via the tail vein. The acquired PET emission image was dynamically rebinned with 20 frames of 20 s, 10 frames of 180 s, 10 frames of 300 s and 5 frames of 400 s using the Siemens Inveon Research Workplace software. The anatomical image acquired from CT was registered with the PET image and the result was represented as %ID/g.

### Cerenkov luminescence imaging

For the *in vivo* experiment, 13 mice were injected with 100 μL of the formulated [^68^Ga]GaCl_3_ (9 to 24 MBq) via the tail vein. Shortly after injection, the mice were anesthetized with an oxygen and isoflurane mixture (3% induction, 2% maintenance) and placed inside the IVIS. The manual focus function of the IVIS was used to focus on the surface of the skin prior to the scan, and a white light image of the mouse was taken. The mice were scanned 45 to 90 min after injection for a duration of 5 min, and only one mouse per scan was done using view A with small binning (4.0 cm × 4.0 cm field of view, 480 × 480 pixels, 0.083 mm × 0.083 mm pixel size, f/stop = f/1).

In order to qualitatively compare blood vessel attenuation of Cerenkov light from [^68^Ga]GaCl_3_, 100 μL (9 to 13 MBq) of [^18^F]FDG was injected into five mice. The mice were not fasted prior to the [^18^F]FDG experiment in order to minimize uptake to the tumor and allow for easier visualization of vasculature. The mice were scanned 60 to 90 min after injection for 20 min, and only one mouse per scan was done using view A with small binning. A 20-min scan duration was used instead of 5 min in order to partially compensate for the low radiance of Cerenkov light for ^18^F.

All analyses of the CLI results were performed using Living Image 4.3.1 software. The percentage of light attenuated by the blood vessels was calculated by dividing the average pixel value within a section of the blood vessel by the average pixel value to the immediate right and left of the blood vessel using the same ROI. The amount of light attenuated by the blood vessels on the tumor side and non-tumor side was evaluated, and the significance was determined using Student’s *t* test (two-tailed, 95% confidence interval).

### Histology

After euthanasia, the skin samples were removed encompassing the tumor and the surrounding skin tissue. The tissue samples were fixed in 4% paraformaldehyde and were sent for histology the following day. Histology work was performed in the Advanced Molecular Pathology Laboratory, Institute of Molecular and Cell Biology, Agency for Science, Technology and Research, Singapore. The wet tissue was sectioned carefully to contain both the tumor and blood vessels. The samples were cut in 5-μm-thick sections, and the slides were stained with hematoxylin and eosin (H&E).

The H&E slides were imaged using a Nikon SMZ18 microscope. The blood vessel diameter was determined by measuring the short axis of the vessels from the digital images taken by the microscope.

## Results

### Phantom results

**Figure 1:**
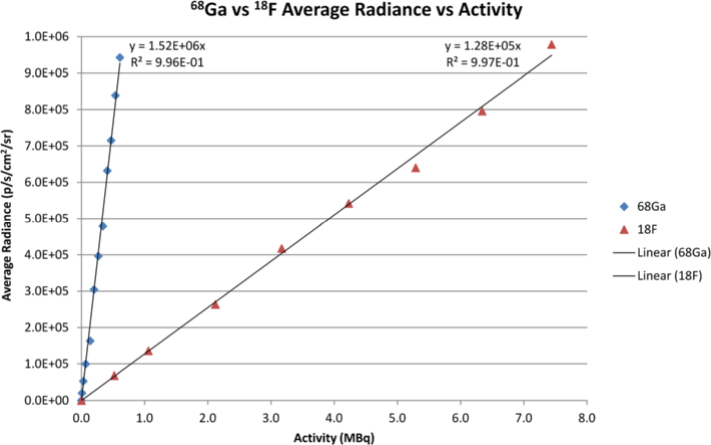
**Plot of radiance in p/s/cm**^**2**^**/sr versus activity in MBq of samples.** Plot of radiance in p/s/cm^2^/sr of samples of varying levels of activity of [^68^Ga]GaCl_3_ (blue) and [^18^F]FDG (red) for a 5-min scan on an IVIS spectrum.

A plot of the radiance per dose is shown in Figure 1. The average radiance of [^68^Ga]GaCl_3_ based on the slope was 1.52 × 10^6^ p/s/cm^2^/sr per MBq with an *R*^2^ value of 0.996. The average radiance of [^18^F]FDG based on the slope was 1.28 × 10^5^ p/s/cm^2^/sr per MBq with an *R*^2^ value of 0.997. Thus, the radiance of ^68^Ga was approximately 11.9 times higher than the radiance of ^18^F.

### Biodistribution results

**Figure 2:**
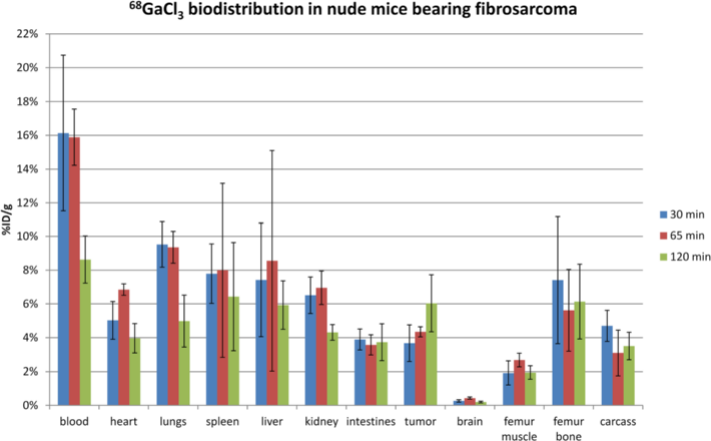
**Biodistribution of [**^**68**^**Ga]GaCl**_**3 **_**in mice 30, 65, and 120 min after injection.** A gamma counter was used (three mice per group).

**Figure 3:**
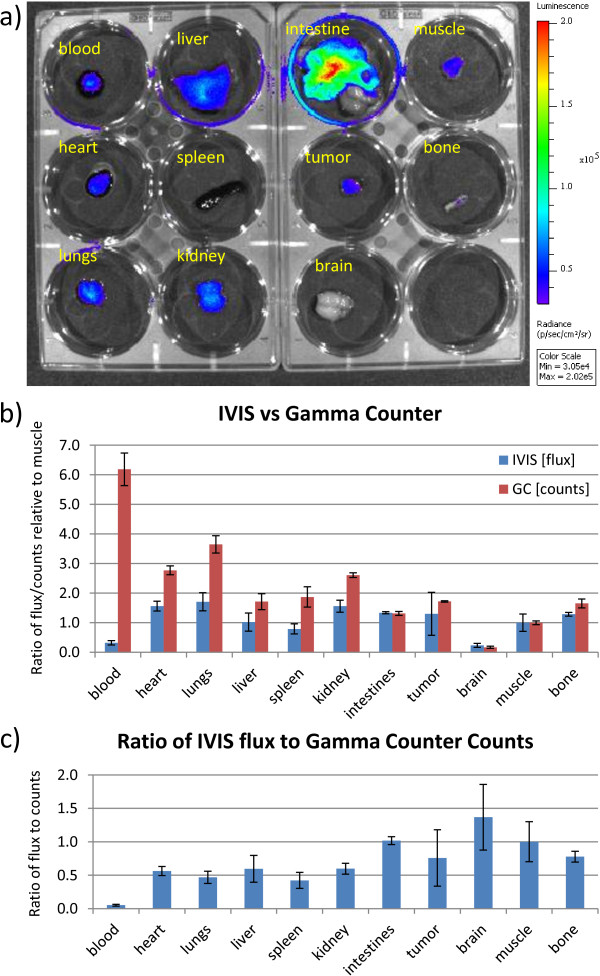
**Comparison of IVIS and gamma counter biodistribution measurements for two of the mice. (a)** The IVIS biodistribution of [^68^Ga]GaCl_3_ in a mouse 65 min after injection. **(b)** Bar graph comparing the IVIS biodistribution in flux (photons/s) versus the gamma counter measurements of the decay activity (gamma ray counts) normalized to the thigh muscle for two mice 65 min after injection. The intestines, tumor, brain, muscle, and femur bone have similar values, but the heart, lungs, spleen, liver, blood, and kidneys show reduced flux relative to the activity. While the blood had the highest activity of 6.2 times muscle, the Cerenkov light output was only 0.32 times muscle. **(c)** Ratio of IVIS flux to the gamma counter counts normalized to muscle. Compared to muscle, the ratio of Cerenkov light to radioactivity was only 0.05, demonstrating that blood attenuated much more light than the muscle and other organs.

A plot of the biodistribution of nude mice taken at 30, 65, and 120 min after injection is shown in Figure 2. The largest amount of radioactivity was found in the blood followed by the liver and spleen. Tumor and intestinal uptake were relatively low with negligible uptake in the brain. At 65 min post-injection, the blood had a %ID/g of 15%, indicating that ^68^Ga remained in the blood long after injection. Even after 120 min, the blood still had the highest %ID/g compared to the other tissues.Figure 3 shows the biodistribution for one of the mice using the IVIS. Figure 3b shows a bar graph comparing the biodistribution of the IVIS versus the gamma counter for two of the mice 65 min after injection with values normalized to the thigh muscle. Figure 3c shows the ratio of IVIS flux to radioactivity as measured by the gamma counter normalized to muscle. The intestines, tumor, brain, muscle, and femur had similar values for flux versus decay activity, while the heart, lungs, spleen, liver, and kidneys had lower flux. The blood had the lowest Cerenkov light output versus decay activity, and the total flux was extremely low despite the blood having the highest decay activity. The ratio of IVIS flux to gamma counter counts was roughly 20 times lower than the same ratio for the thigh muscle, indicating high attenuation of Cerenkov light by the blood.

### *In vivo* results

**Figure 4:**
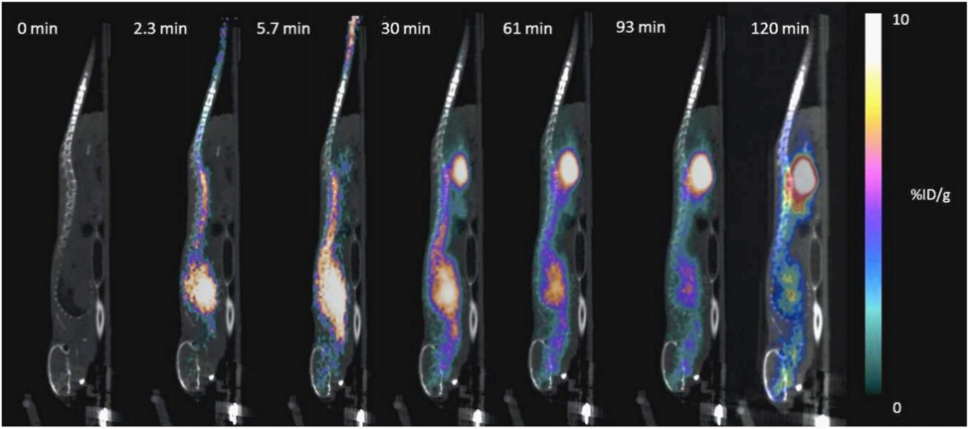
**PET/CT images of [**^**68**^**Ga]GaCl**_**3 **_**distribution in nude mice shown from 0 to 120 min post-injection.** The radiotracer was observed to be distributed throughout the body and cleared through the renal system. The large signal from the heart indicates that much of the radiotracer remained in the blood.

Figure 4 shows PET/CT images of [^68^Ga]GaCl_3_ from 0 to 120 min after injection. In the first few minutes after injection, the radiotracer signal was observed to be highest from the heart. After 30 min, the tracer was distributed throughout the body with clearance through the renal system. The signal from the heart remained high for the entire 120-min duration due to the circulation of ^68^Ga in the blood.

**Figure 5:**
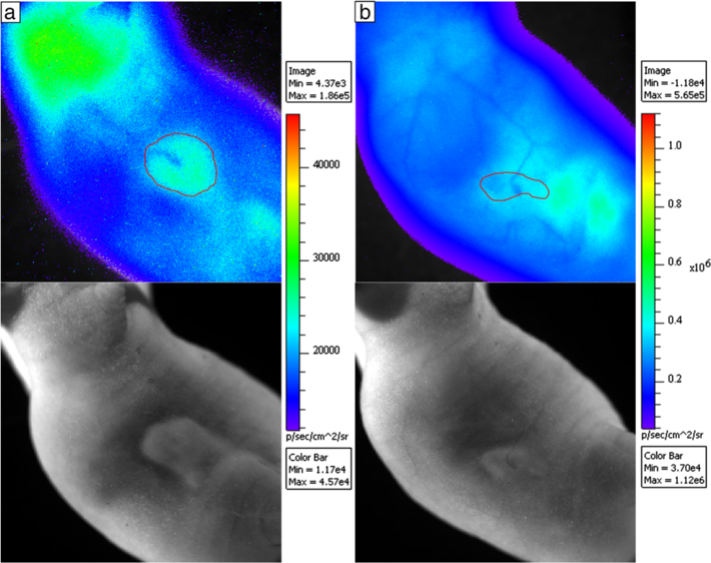
**Comparison of [**^**18**^**F]FDG and [**^**68**^**Ga]GaCl**_**3 **_**imaging of the vasculature in mice. (a)** CLI using [^18^F]FDG on a day-14 fibrosarcoma tumor with corresponding photographic image below. **(b)** CLI using [^68^Ga]GaCl_3_ for a day-14 fibrosarcoma tumor with corresponding photographic image below. The tumors are outlined in red. A blood vessel can be seen in the [^18^F]FDG image, resulting in a 6% reduction in radiance outside the tumor and 12% reduction in the tumor core. However, the vasculature is much clearer and more elaborate in the [^68^Ga]GaCl_3_ image, where the blood vessel in the tumor attenuated 9% of the Cerenkov light and the tumor core attenuated 18% of the Cerenkov light.

Figure 5 shows a comparison of [^18^F]FDG and [^68^Ga]GaCl_3_ imaging of the vasculature in mice at day 14 post-injection of tumor cells. Figure 5a shows CLI of a mouse with the corresponding photographic image below 75 min after injection with 13 MBq of [^18^F]FDG, and Figure 5b shows CLI of a mouse with the corresponding photographic image below 50 min after injection with 14 MBq of [^68^Ga]GaCl_3_. The activities at the time of each scan were 8.1 MBq for [^18^F]FDG and 8.4 MBq for [^68^Ga]GaCl_3_, and the total flux from each mouse was 3.4 × 10^6^ p/s and 4.0 × 10^7^ p/s, respectively. Thus, the amount of light produced per megabecquerel was 12.2 times higher for ^68^Ga than ^18^F, matching the phantom measurements. Due to the high photon attenuation of Cerenkov light of the blood, the blood vessels were clearly observed in both scans, with a 6% reduction in radiance due to the blood vessel in the [^18^F]FDG scan and 9% reduction in radiance by the blood vessel entering the tumor in the [^68^Ga]GaCl_3_ scan. Moreover, a 12% reduction in radiance was observed at the tumor core of the [^18^F]FDG scan, and an 18% reduction in radiance was seen at the tumor core in the [^68^Ga]GaCl_3_ scan. Qualitatively, extensive vasculature including major blood vessels and branching of the vessels could be observed in the [^68^Ga]GaCl_3_ scan, whereas only a single blood vessel could be seen in the [^18^F]FDG scan.

**Figure 6:**
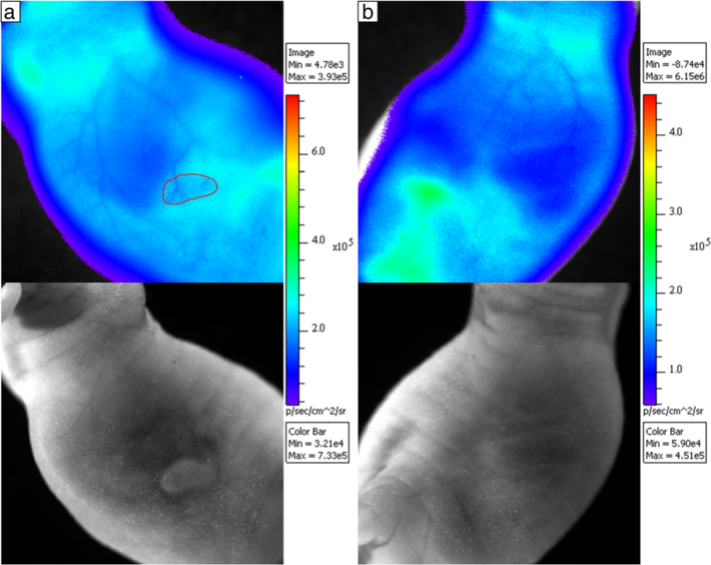
**Tumor side and non-tumor side CLI with corresponding photographic images.** CLI using [^68^Ga]GaCl_3_ on a day 16 fibrosarcoma tumor on **(a)** tumor side and **(b)** non-tumor side with corresponding photographic images below. The vasculature on the tumor side is more prominent, with the lateral vessel entering the tumor attenuating 8% of the Cerenkov light, whereas the lateral vessel on the non-tumor side attenuated only 3% of the Cerenkov light. The tumor is outlined with red enclosure.

**Figure 7:**
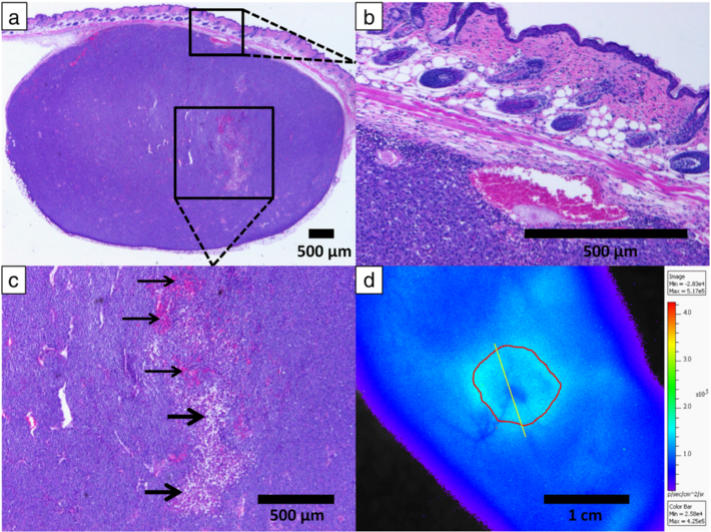
**H&E stain and CLI of a 21-day post-injection fibrosarcoma tumor. (a)** H&E stained histology sample showing the tumor with necrotic core and large blood vessel. **(b)** H&E stained histology sample showing a magnified section of a large subcutaneous blood vessel. **(c)** H&E stained histology sample showing a magnified section of the necrotic core. The accumulation of red blood cells within the necrotic core is indicated by the thin arrows, while the white spaces showing the location of cell necrosis are indicated by the thick arrows. **(d)** CLI of the mouse from where the H&E stain was taken as indicated by the yellow line with the tumor outlined in red. The necrotic core and the large blood vessel entering the tumor are clearly seen with 21% and 12% reduction in radiance, respectively.

Figure 6 shows the tumor side and non-tumor side with corresponding photographic images below for a mouse 90 min after injection with 20 MBq of [^68^Ga]GaCl_3_. Although vasculature was visible on the tumor and non-tumor sides of the animal, the amount of light attenuated was much higher on the tumor side. The blood vessel on the tumor attenuated 8% of the Cerenkov light, whereas the non-tumor side blood vessel attenuated only 3% of the Cerenkov light.Figure 7 shows an H&E stain and CLI of a 21-day post-injection fibrosarcoma tumor, which had a blood vessel leading into the core of the tumor similar to Figure 5. The mouse was injected with 10 MBq of activity, and the mouse was scanned at 90 min after injection. Figure 7a shows a cross section of the tumor with H&E stain with the major blood vessel and necrotic core visible. Figure 7b shows a magnified image of a subcutaneous blood vessel in the tumor, which appears dilated due to the presence of the tumor. Figure 7c shows a magnified image of the necrotic region with thin arrows indicating areas of high accumulation of red blood cells and thick arrows indicating areas of necrosis. Figure 7d shows the CLI of the mouse where the histology section was taken. The section taken is indicated by the yellow line, and the outline of the tumor is shown in red. The necrotic core is clearly seen in the image with a 21% reduction in radiance, where the accumulation of red blood cells in the necrotic area caused high attenuation of the Cerenkov light. Figure 7d also shows the dilated tumor blood vessel from Figure 7b, which attenuated 12% of the Cerenkov light.

**Figure 8:**
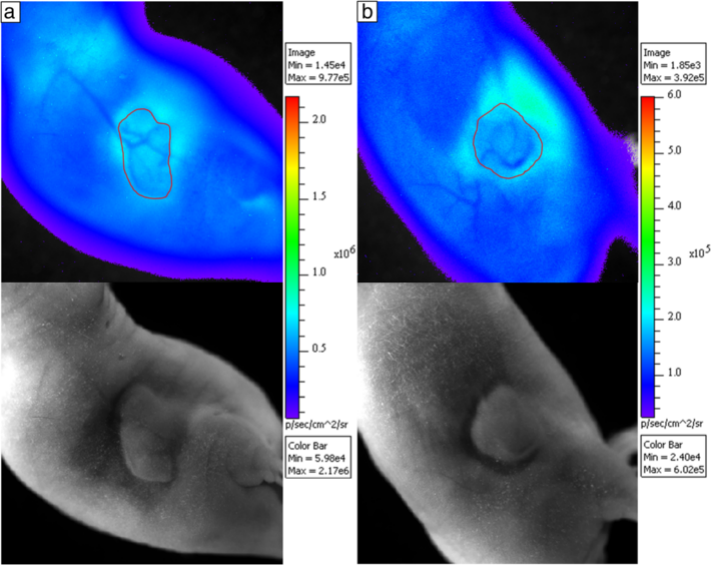
**CLI of highly vascularized tumors with prominent blood vessels.** CLI using [^68^Ga]GaCl_3_ for subcutaneous fibrosarcoma models at day 21 below with hypervascularization of tumors (outlined in red) with scans taken **(a)** 45 min after injection of 24 MBq and **(b)** 60 min after injection of 11 MBq with corresponding photographic images below.

Figure 8 shows highly vascularized tumors with prominent blood vessels. Figure 8a shows CLI with corresponding photographic image below of a mouse 45 min after injection with 24 MBq of [^68^Ga]GaCl_3_, and Figure 8b shows CLI with corresponding photographic image below of a mouse 60 min after injection with 11 MBq of [^68^Ga]GaCl_3_.

For the 13 mice scanned, the tumor and non-tumor sides were imaged and the attenuation of light from the blood vessels was measured. The tumor blood vessels attenuated 7% to 18% of the light for an average attenuation of 11% ± 4%, whereas the lateral vein on the non-tumor side of the mouse running the length of the body attenuated 2% to 4% of the light for an average attenuation of 3% ± 1%. The difference was significant with a *p* value of 1.0 × 10^−5^.

## Discussion

Using the negative contrast imaging technique, it was possible to use CLI to image the blood vessels in nude mice due to the high absorption of red and infrared light by the red blood cells compared to the surrounding tissue [19]. We observed in Figure 3 that different organs had different light attenuation properties, and the blood had particularly high photon attenuation. Our results suggest that the red blood cells attenuate the light resulting in reduced signal in the blood vessels and the necrotic area of the tumor as seen in Figure 7d. Although the blood contains a large proportion of the radiotracer (6.2 times muscle), Figure 3 shows that the resulting Cerenkov light from the blood is quite low (0.32 times muscle). Thus, the blood vessels are visualized since the combined Cerenkov light output of all deeper tissues results in a full body backlighting, which is attenuated by the blood in the blood vessels before reaching the camera, resulting in a negative contrast. The tumor blood vessels attenuate more light than non-tumor blood vessels primarily due to the larger diameter of the dilated vessels, which results in greater attenuation due to the increased amount of blood the light must travel through.

In this paper, we used [^68^Ga]GaCl_3_ as a CLI tracer due to its characteristically high light output and long blood circulation time. When injected intravenously, [^68^Ga]GaCl_3_ provided background illumination that allowed for visualization of the anatomical structures that had higher attenuation of light than surrounding tissues. We propose that visualization of blood vessels is possible since blood attenuates more Cerenkov light than the surrounding tissue. Cerenkov light is emitted at all frequencies in the visual light range but produces more photons at shorter wavelengths of light, resulting in the characteristically blue color of Cerenkov light. Since blue light is greatly attenuated by biological tissues, Cerenkov light emitted from mice injected with [^68^Ga]GaCl_3_ will mostly consist of longer wavelength photons such as red and infrared light. Since the blood, and especially venal blood, attenuates more light in the red region than surrounding tissue [20], negative contrast of blood vessels is possible.

For our study, we used HT-1080 fibrosarcoma tumors for imaging blood vessels, which greatly expresses VEGF/VPF under both normoxic and hypoxic conditions [21]. Angiogenesis observed in solid xenograft tumors leads to co-option of neighboring tissues, resulting in dilation of existing normal vessels [22-24]. The dilation of these blood vessels is an important consequence of tumor development which is primarily absent on the contralateral (i.e., non-tumor) side. CLI provides visualization of such vessels as a result of attenuation of the background-illuminated Cerenkov radiation. Additionally, the necrotic core of tumors, typically characterized by dead tissue due to hypoxic conditions [25], has greater attenuation of light compared to surrounding tumor tissue primarily due to the accumulation of blood in the necrotic regions as seen in Figure 7c.

While blood vessel imaging using negative contrast CLI could possibly be performed with other radiotracers, [^68^Ga]GaCl_3_ was optimal for blood vessel imaging for a few reasons. One reason is that ^68^Ga has very high light output with nearly 12 times the light output of ^18^F, which allowed for imaging with high sensitivity. As seen in Figure 5, the blood vessel light attenuation was observed using both [^68^Ga]GaCl_3_ and [^18^F]FDG, but the image quality was much better using [^68^Ga]GaCl_3_ with much more extensive vasculature visible. The poor image quality of [^18^F]FDG was likely due to the greatly reduced radiance that could only be partially compensated with longer exposure time (20 min vs. 5 min). Using the same imaging parameters as [^68^Ga]GaCl_3_ (5 min exposure and small binning) resulted in no discernible blood vessels when [^18^F]FDG was injected. The data suggest that other radiotracers with high Cerenkov light output, such as ^90^Y and ^32^P, could also be used for negative contrast imaging of the blood vessels. Another reason for using [^68^Ga]GaCl_3_ for blood vessel imaging is that the radiotracer remains in the blood for a long period after injection as observed in the biodistribution shown in Figure 2. Moreover, the dynamic PET/CT in Figure 4 shows a high signal in the heart even though the biodistribution shows fairly low uptake in the heart, so it can be concluded that ^68^Ga stayed in the blood with minimal uptake in the myocardium. Since light is scattered and attenuated by the tissue, the radiance at the surface as observed by the IVIS camera was fairly evenly distributed even though the distribution observed using PET was not as uniform. The distribution of radiotracer allowed for an adequate amount of light to visualize blood vessels near the surface of the skin from the neck to flank.

There are a number of factors to consider when performing negative contrast CLI of blood vessels. One factor is the source of Cerenkov light providing the backlighting necessary for negative contrast imaging of the blood vessels. Since the radiotracer is distributed throughout the body, light is emitted from all directions relative to the blood vessels. That means that superficial blood vessels, which are typically 0.2 to 0.4 mm under the surface of the skin, can be poorly delineated due to both light emitted from the radiotracer and the scattering of light between the blood vessel and the surface of the animal. As a result, the objects at the surface of the animal, such as fur, are clearly delineated, whereas deeper blood vessels cannot be visualized at all. With the most optimal focusing, the width of the blood vessels in the CLI images was about 0.4 mm, which was approximately 10 times the actual blood vessel diameter. Respiration was also a factor in image quality. Although mice were positioned so that motion in the *x* and *y* directions was minimized, respiratory motion in the *z* direction caused the skin and blood vessels to move in and out of focus resulting in blurring. One way to minimize the blurring effect of respiration is to reduce the aperture size (i.e., f/stop). Reducing the aperture size from f/1 to f/2 can provide sharper images of the blood vessels by allowing for a greater depth of focus. However, a reduction of f/stop from f/1 to f/2 results in a 4-time reduction in the signal, which requires a longer exposure time. Thus, it was determined that there was little qualitative improvement in reducing the aperture size since the blood vessels will be in focus for most of the respiratory cycle, so signal was maximized by choosing an f/stop of f/1.

There are some potential applications for imaging vasculature with CLI. The angiogenic behavior of tumors is a highly researched topic [26], and imaging of the blood vessels near the surface has been proven useful using techniques such as intravital microscopy [27]. Other potential applications that our research has demonstrated are the use of negative contrast CLI to detect necrosis within subcutaneous tumors and measurements of the dilation of tumor blood vessels. Although the blood vessels could be seen with the naked eye as well as in the photographs, CLI has the potential advantage of quantifying blood vessel dilation through measurements of the attenuation of Cerenkov light by the blood vessel.

Figure 6 demonstrates the advantage of using CLI for imaging blood vessels because CLI could not only image both tumor and non-tumor blood vessels, but it could also measure the difference via attenuation of Cerenkov light. This study consistently showed higher light attenuation from blood vessels in or on the tumor than blood vessels on the non-tumor side. Whereas typical subcutaneous blood vessels are 10 to 50 μm in diameter [28,29], tumor blood vessel diameters can exceed 100 μm, which was the case for the mouse in Figure 7. The dilated tumor blood vessels attenuated more Cerenkov light than the non-dilated, non-tumor side blood vessels since Cerenkov light passes through more blood in the larger diameter blood vessels. It was observed that tumor vessels attenuated approximately three times more light at 10% ± 4% than the non-tumor side vessels at 3% ± 1%. Qualitatively, the blood vessels on the tumor side were much clearer and easier to delineate than the non-tumor side blood vessels.

The photon attenuation of blood vessels has been observed in other techniques, such as intravital microscopy, and the absorption properties of blood are well-known [20]. The high photon absorption of the blood resulted in greater attenuation in the blood vessels than the surrounding tissue, which allowed for imaging of blood vessels and areas of blood accumulation. For example, histology confirmed red blood cell accumulation inside the tumor in Figure 7c with a 21% reduction of radiance at the tumor core as shown in Figure 7d. Thus, CLI could identify necrosis within the tumor since accumulation of red blood cells is one of the characteristics of a necrotic core [30]. The reason for the lack of positive signal from the blood vessels despite the presence of ^68^Ga in the blood was that the amount of radiance caused by the positron decays of ^68^Ga was insignificant compared to the high attenuation of Cerenkov light in the blood as observed in Figure 3. The cumulative effect of all photons emitted from internal tissues created a whole body internal illumination that acted as a system of background lighting, which allowed for negative contrast imaging of superficial structures such as the blood vessels.

## Conclusions

Using [^68^Ga]GaCl_3_, it was possible to image vasculature in mice with CLI. The high light output and the distribution of [^68^Ga]GaCl_3_ within the body make the radiotracer an excellent tool for blood vessel imaging. Negative contrast imaging is different from conventional CLI techniques, which typically look at areas of high uptake of the radiotracer similar to other nuclear medicine techniques. By taking advantage of the optical properties of CLI, a new approach of negative contrast imaging can be done. Not only is it possible to image the distribution of the radioisotope, but it is also possible to image subcutaneous blood vessels. Our data show that imaging of blood vessels is feasible using a radiotracer with high Cerenkov light output, which could potentially lead to the development of CLI for angiography.

## Competing interests

The authors declare that they have no competing interests.

## Authors’ contributions

JDS, AR, and PC were involved in the study design, implementation, analysis, and manuscript preparation. JPA, EGR, and DWT were involved in the study design and manuscript preparation. KK and CTY were involved in the implementation of the study and manuscript preparation. All authors read and approved the final manuscript.
